# Model of Carbon Footprint Assessment for the Life Cycle of the System of Wastewater Collection, Transport and Treatment

**DOI:** 10.1038/s41598-020-62798-y

**Published:** 2020-04-02

**Authors:** Paweł Zawartka, Dorota Burchart-Korol, Agata Blaut

**Affiliations:** 1 Central Mining Institute, Plac Gwarków 1, 40-166 Katowice, Poland; 2 Silesian University of Technology, ul. Krasińskiego 8, 40-019 Katowice, Poland

**Keywords:** Climate-change impacts, Environmental impact

## Abstract

This article presents a model of the environmental assessment of the system of wastewater collection, transport and treatment. The model was developed based on an original environmental assessment method of a system consisting of four elements: septic tanks, household wastewater treatment plants, a sewerage system and a central wastewater treatment plant. To conduct the environmental assessment, the Life Cycle Assessment technique was applied. The Intergovernmental Panel on Climate Change (IPCC) method was also applied, which enabled the determination of the carbon footprint of the analysed wastewater management system. This article presents the outline of an original method applied to create a model and an inventory of the data necessary for environmental assessment and the application of the model for the environmental assessment of a system of wastewater collection, transport and treatment in a city with over 50.000 inhabitants. Three feasible variants (from a functional, technical, organizational and financial point of view) of the system’s development were analysed. The variants were subjected to comparative analysis using the solution. The obtained results, together with the assessment method can be used as a practical tool to assess whether the European Commission’s guidelines are met, as well as the challenges facing wastewater management in the circular economy are overcome.

## Introduction

Steps taken in the area of environmental protection ought to follow the principle of sustainable development which is understood as “… meeting the needs of the present without compromising the ability of future generations to meet their own needs”^1^. From the point of view of implementing the rules of sustainable development, it is crucial to create stable systems of wastewater collection and treatment, which are friendly for the society, environment and economy^2,3^. Due to increasing requirements concerning soil and water environment protection, the issues of efficient wastewater collection, transport and treatment still remain a challenge. It is particularly important in areas subjected to urbanization and in rural areas where the issue of wastewater treatment requires active steps in order to address it and limit its negative influence on the environment^4^. Due to a wide range of available technological solutions, it is necessary to consider different system solutions, both centralised ones and decentralised ones^5^ The selection of an optimal solution is challenging and it is usually influenced by certain conditions it has to meet, i.e. minimal cost of construction, minimal sewerage charge, following environmental protection regulations, or improving the standard of living of the users^6^.

The selection of a wastewater management method in order to minimize the influence on the environment is a rare situation and it is most often caused by specific local conditions, as well as detailed requirements imposed by environmental protection authorities or arrangements made by public consultations^7^. The conditions shaping wastewater management are complex and cost-intensive and the long process of implementing wastewater management systems ought to be preceded by an in-depth analysis concerning the selection of the optimal solution^5^.

Legal and administrative, economic, and environmental conditions determine wastewater management. Fundamental problems in wastewater management are a lack of knowledge on the subject and a lack of methods and comparative tools for the complex analysis of the environmental impact of solutions for both central wastewater treatment plants and household wastewater treatment plants^7^. This article addresses this gap by presenting a model of the environmental assessment of a system of wastewater collection, transport and treatment which consists of: a sewerage system, a central wastewater treatment plant, septic tanks and household wastewater treatment plants. Each element in this system can be assessed separately, but it is also possible and reasonable to assess the system as a whole. Life Cycle Assessment (LCA), and carbon footprint analysis to be precise, was selected as the environmental assessment methodology. LCA is considered to be a complex research method for environmental assessment as it enables the influence of life cycle stages of a product, i.e. its construction, use, end-of-life, on the environment to be studied. This method was used in many fields: e.g. mining^8^, steel production^9^, logistic^10^, energy production^11^, etc.

Despite widespread use of the method, research on the application of the environmental LCA in sewerage systems which have been published so far most often only concern wastewater treatment plants without the wastewater transporting system. The results of environmental analyses conducted with the LCA method particularly focus on comparisons of different treatment technologies from an environmental point of view^12^. With regards to wastewater sludge processing and management, the results focus on its disposal, recycling and circulation in wastewater treatment installations^13–16^.

With reference to the sewerage system, the literature mainly provides examples of employing LCA to assess the environmental influence of a pipeline built from different materials^17,18^. This is partially a consequence of the fact that sewerage system networks were not the centre of attention of LCA analyses, and they were treated as an unimportant, and hence negligible, element of the existing wastewater management system.

LCA is the most common method for evaluating environmental impact which enables a complex analysis of all the elements of a wastewater treatment system^19,20^. The negative influence of septic tanks on the soil and water environment is described in many foreign publications^21–24^. Opher and Friedler^25^ performed a Life Cycle Assessment to compare the environmental impacts assessment of alternatives for a city’s water-wastewater service system. It was concluded that a decentralized approach to urban wastewater management is environmentally preferable to the common centralized system. The state of the art of greenhouse gases from wastewater treatment plants was done by Mannina *et al*.^26^. Basing on previous LCA analyses for wastewater treatment plants, it has been emphasized that it is necessary to unify and standardize the methodology of LCA application for wastewater treatment systems. Methodology for the selection of sustainable sewerage servicing systems and technologies was presented in the study of Sharma *et al*.^27^. A comparative LCA of vertical flow constructed wetlands and horizontal flow constructed wetlands was carried out by Fuchs *et al*.^28^. It was concluded that gaseous emissions, often not included in wastewater LCAs because of a lack of data. In the study of LCA of alternative wastewater treatment processes^29^, it was concluded that the LCA approach can be used as a decision making tool in design studies, but information for environmental impact assessment and minimization measures requires further improvement.

Corominas *et al*.^12^, based on the critical review of previous LCA analyses conducted for wastewater treatment plants, underlined the need to unify and standardise the methodology of the LCA for wastewater treatment systems. The critical issue is the determination of a quantifiable functional unit against which the LCA analysis is conducted. In previous publications functional units were assumed arbitrarily, which makes it impossible to compare their results.

The LCA analysis conducted focused on greenhouse gas emission. This emission is a very important and popular topic now^30^. The literature review showed that the issues associated with greenhouse gas emission from the system of wastewater collection, transport and treatment were analysed for a short period time. Wastewater treatment plants are the source of greenhouse gas emission, i.e.: carbon dioxide (CO_2_), nitrous oxide (N_2_O) and methane (CH_4_) which result from biological processes occurring in wastewater.

The methodology of quantitative greenhouse gas emission (GHG) from wastewater and sewerage systems was developed by the Intergovernmental Panel on Climate Change (IPCC)^31^. The calculations presented in the European Commission’s Report, concerning gas emission from sewerage systems, based on the developed methodology, show that 9% and 3% of world emission of CH_4_ and N_2_O, respectively, comes from wastewater treatment plants. The report showed that the data may be underestimated, as gas emission forecasts often omit the gas emission which occurs while transporting wastewater to a wastewater treatment plant, as well as the gas emissions from scattered objects and installations for collecting and treating wastewater.

To conduct an environmental assessment with a Life Cycle Assessment of the system of wastewater collection, transport and treatment, as well as the European Commission’s latest guidelines and recommendations concerning the assessment of the influence of enterprises, and especially their greenhouse gas emission, on the climate, this study presents an assessment of GHG emission in the analysed system.

There are publications in the literature that present the results of LCA analyses of individual system elements, however, there are no publications which present a computational model for performing the carbon footprint of the life cycle assessment of a system.

The goal of this study was to develop a computational life cycle of greenhouse gas emission in order to assess the environmental impact of the system of wastewater collection, transport and treatment. The objective of this paper was also the application of an LCA model to calculate the carbon footprint of the system at a national level.

## Material and methods

The aim of the research was the development of a computational model which would be used to assess environmental impact throughout the life cycle (construction, use and end-of-life stages) of a system of wastewater collection, transport and treatment. In their article Burchart-Korol *et al*.^32^ presented a similar LCA model, but about coal mine operations. The wastewater management model was prepared based on a complex inventory of the inputs and outputs of the analysed system, which specified determinants of the influence on the environment.

The developed model of the environmental Life Cycle Assessment of the system of wastewater collection, transport and treatment includes four elements – septic tanks, household wastewater treatment plants, a sewerage system and a central wastewater treatment plant. The model enables the calculation of the aggregated influence of the system or its elements on the volume of greenhouse gas emission throughout the life cycle or at during its different stages, ie. construction, use and end-of-life, for a given technical configuration of the system and a given size of the system expressed with the total Person Equivalent (PE). Attributional life cycle assessment (LCA) was used to assess the potential environmental burdens. Greenhouse gas emission assessment was performed using the identified main sources of environmental indicators, using the SimaPro v. 8 package with the Ecoinvent v.3 database

- The life cycle assessment was conducted in accordance with ISO 14044:2006^33^. The LCA consisted of four steps:
- Goal and scope definition: The purpose of the study, the functional unit, system boundaries and association were defined.
- Life Cycle Inventory (LCI): In the LCI relevant input and output data was collected and quantified.
- Life Cycle Impact Assessment (LCIA): Environmental indicators were calculated according to the IPCC method
- Interpretation.

**Figure 1:**
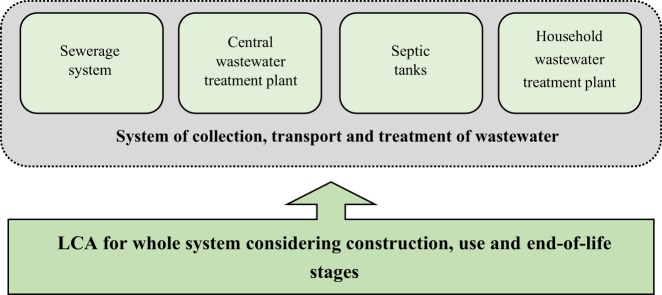
The scope of the LCA of the system of collection, transport and treatment of wastewater.

The scope of the LCA model includes four elements – septic tanks, household wastewater treatment plants, a sewerage system and a central wastewater treatment plant within the life cycle (construction, use, end-of-life stages) of the system of collection, transport and treatment of wastewater (Fig. 1).

**Figure 2:**
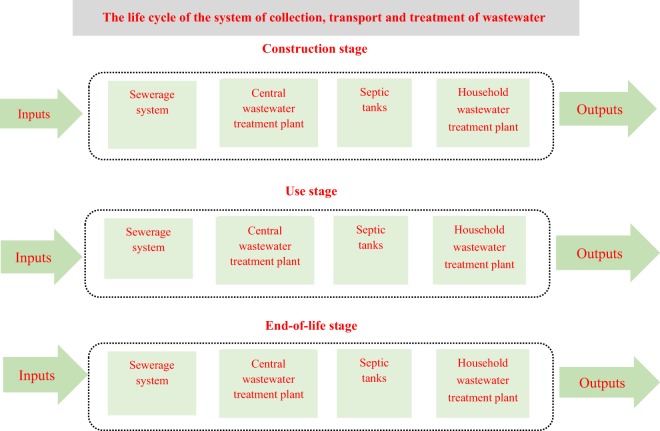
System boundary of the life cycle of the system of collection, transport and treatment of wastewater. Source: Own research.

System boundaries were determined and inputs and outputs for a given life cycle stages were identified for the life cycle of each element of the system of wastewater collection, transport and treatment system. The system boundaries are presented in Fig. 2.

The environmental assessment was conducted with the Life Cycle Assessment (LCA) technique applying the Intergovernmental Panel on Climate Change method (IPCC), i.e. following the methodology proposed by the Intergovernmental Panel on Climate Change. The carbon footprint index, which is a result of applying the method, is calculated based on global warming potential (GWP) and is expressed in CO_2_ equivalent. The greenhouse gas emission indices necessary for the analysis were taken from the Ecoinvent 3 database. The results are presented as greenhouse gas emission expressed in CO_2_ equivalent with reference to a functional unit, which is 1 Person Equivalent (1PE) served by the system. This functional unit allows a comparative analysis between objects and it is universal for every municipal sewage system whilst taking into consideration its diversity The adopted unit is the foundation to create a universal model to assess water and sewage management. 1 PE has already been used as a functional unit in the LCA technique in Corominas *et al*.^12^. The functional unit of the analysis is 1 PE as a parameter which is universal for each urban wastewater system whilst taking into consideration its diversity. The assumed functional unit enables comparative analyses with other facilities and is the foundation for the creation of universal models to assess water and sewage management.

**Figure 3:**
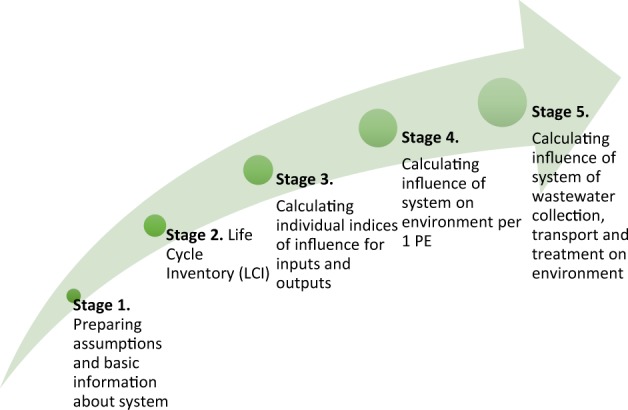
Five-stage environmental assessment method of a system consisting of four elements: septic tanks, household wastewater treatment plants, sewerage system and central wastewater treatment plant. Source: Own research.

To prepare a model of carbon footprint assessment of the system of wastewater collection, transport and treatment, an original method consisting of five stages was employed, these being: preparing assumptions and basic information about the system, Life Cycle Inventory (LCI), calculating individual influence indices for inputs and outputs, calculating the influence of the system on the environment per 1 PE, and calculating the influence of the system of wastewater collection, transport and treatment on the environment (Fig. 3).

### Stage 1. - Preparing assumptions and basic information about the system

The work carred out in the development of the LCA model included the steps described below. The first step was the identification of the input and output for each element (LCI for septic tanks, household wastewater treatment plants, sewerage system and central wastewater treatment plant) within the life cycle based on information collected from the system of wastewater collection, transport and treatment in Poland.

Within the first stage, the following assumptions and basic information about the system were prepared:

- data on the number of users of each of the elements of the system expressed in PE – septic tanks, household wastewater treatment plants, a sewerage system and the central wastewater treatment plant,
- data on septic tanks – number, material, average capacity, qualitative and quantitative parameters of the transported wastewater,
- data on household wastewater treatment plants – number, type, treatment efficiency, qualitative and quantitative parameters of transported sludge,
- data on the existing and/or designed sewerage system – length, material, diameters, number of wastewater pumping stations,
- data on the wastewater treatment plant – assumed load of contaminants expressed in PE, the volume of wastewater treated per year, electricity and heat production from biogas (YES/NO), share of energy produced from biogas in the total energy used, concentration of treated wastewater discharged from a wastewater treatment plant, the level of reduction of pollutants,
- supplementary data – use of water in the system, the average distance between the tanks, household wastewater treatment plants (HWTP) and the central wastewater treatment plant.

Detailed description of the life cycle of the system of wastewater collection, transport and treatment system was presented by Zawartka^34^. The data concerned the differentiation of each system of wastewater collection, transport and treatment, hence applying the Life Cycle Assessment method requires the individual determination of the assumptions and the provision of given information.

### Stage 2. – Life cycle inventory (LCI)

By applying the collected data to the system, the next stage was the detailed inventory of the data concerning materials used, energy and substances, and pollution emission into the environment at given life cycle stages for each of the elements of the system. The inventory enabled, at further stages, calculation of the greenhouse gas emission index.

To prepare the assessment model, the following notations (used in computational formulas) were assumed:

- Index ‘i’ represents given types of materials used, energy and substances, and pollution emission into the environment,
- Index ‘j’ represents individual elements of an analysed system: septic tanks, household wastewater treatment plants, a sewerage system and a central wastewater treatment plant,
- Index ‘k’ represents given life cycle stages, including construction, use, and end-of-life stages,

The use of materials, energy and substances, and emissions into the environment were balanced referring to the assumed life cycle of the system (30 years), and then converted referring to the assumed functional unit (PE).

Tables 1–4 contain inventory data with reference to 1 PE (1 Person Equivalent) split into elements of the analyzed system and given life cycle stages.

**Table 1:** Data inventory of septic tanks with reference to a functional unit (1 PE).

Inputs and outputs	Unit	Septic tanks
**CONSTRUCTION STAGE**		
Gravel	kg	379.24
Concrete	m^3^	0.83
Steel	kg	38.93
Cast Iron	kg	15.13
PCV	kg	8.27
Asphalt Rubber	kg	6.66
Polyester Resin	kg	7.16
PE	kg	13.68
Earthworks	m3	9.76
Water	kg	2333.33
**USE STAGE**		
Diesel Fuel	kg/year	92.96
CO_2_ Emission	kg/year	11.05
CH_4_ Emission	kg/year	0.65
N_2_O Emission	kg/year	0.01
Electricity From Grid	kWh/year	68.15
Electricity From Biogas - Production	kWh/year	69.15
Electricity From Biogas - Avoided Electricity From Grid	kWh/year	69.15
**END-OF-LIFE STAGE**		
Recycling-Cast Iron	kg	15.13
Earthworks	m3	3.33

Source: Own research.

**Table 2:** Data inventory of household wastewater treatment plants with reference to a functional unit (1 PE).

Inputs and outputs	Unit	Household wastewater treatment plants
**CONSTRUCTION STAGE**		
Gravel	kg	1329.21
Concrete	m^3^	0.15
Cement	kg	127.57
Stainless Steel	kg	0.49
Cast Iron	kg	2.29
Copper	kg	0.03
PCV	kg	14.78
PE	kg	34.38
Polypropylene	kg	2.15
Earthworks	m3	7.98
Geotextile PP	kg	0.18
Water	kg	761.30
Clay	kg	32.02
**USE STAGE**		
Diesel Fuel	kg/year	48.77
CO_2_ Emission	kg/year	11.05
CH_4_ Emission	kg/year	0.65
N_2_O Emission	kg/year	0.01
Electricity From Grid – Use Of Electricity By Wastewater Treatment Plants 2	kWh/year	9.32
Electricity From Grid - Use Of Electricity For Sludge Treatment 2	kWh/year	0.52
Electricity From Grid	kWh/year	0.00
Electricity From Biogas - Production	kWh/year	9.46
Electricity From Biogas - Avoided Electricity From Grid	kWh/year	9.46
Heat From Biogas - Production	MJ/Year	0.00
Gravel	kg	28.38
Geotextile PP	kg	0.01
Stainless Steel	kg	0.02
Copper	kg	0.00
PCV	kg	0.12
**END-OF-LIFE STAGE**		
Recycling-Cast Iron	kg	2.30
Recycling-Steel	kg	0.49
Recycling-Copper	kg	0.03
Earthworks	m^3^	1.60

Source: Own research.

**Table 3:** Data inventory of a sewerage system with reference to a functional unit (1 PE).

Inputs and outputs	Unit	Sewerage System
**CONSTRUCTION STAGE**		
Gravel	kg	851.25
Concrete	m^3^	0.19
Polymer Concrete	kg	3.16
Stone	kg	49.99
Stoneware	kg	40.66
Steel	kg	0.46
Stainless Steel	kg	0.05
Galvanised Steel	kg	0.50
Cast Iron	kg	15.17
Copper	kg	0.01
PCV	kg	14.09
Synthetic Rubber	kg	0.05
Asphalt	kg	385.57
PE	kg	1.83
Earthworks	m3	12.43
Water	kg	294.87
**USE STAGE**		
Diesel Fuel	kg/year	1.99
Petrol	kg/year	0.03
Electricity From Grid	kWh/year	120.20
Replacement – Sewerage System	kg	252.94
**END-OF-LIFE STAGE**		
Recycling-Cast Iron	kg	15.17
Recycling-Steel	kg	0.58
Recycling-Copper	kg	0.01

Source: Own research.

**Table 4:** Data inventory of a central wastewater treatment plant with reference to a functional unit (1 PE).

Inputs and outputs	Unit	Central Wastewater Treatment Plant
**CONSTRUCTION STAGE**		
Concrete	m^3^	1.80
Aggregate	kg	38.75
Reinforcing Steel	kg	139.96
Stainless Steel	kg	11.23
Cast Iron	kg	0.09
Aluminium	kg	1.56
Copper	kg	1.66
Mineral Wool	kg	1.57
Glass fibre	kg	3.53
Synthetic Rubber	kg	1.59
Asphalt	kg	0.90
PE	kg	4.41
Polyethylene LDPE	kg	0.03
Plastic Foil	kg	4.43
Earthworks	m3	6.28
Water	kg	219.72
Organic Chemicals	kg	7.32
Inorganic Chemicals	kg	0.90
Electricity From Grid	kWh	0.07
**USE STAGE**		
Diesel Fuel	kg/year	0.07
Petrol	kg/year	0.01
Consumables -WWTP PIX/PAX	kg/year	53.26
CO_2_ Emission	kg/year	432.04
CH_4_ Emission	kg/year	1.71
N_2_O Emission	kg/year	0.10
Electricity From Grid	kWh/year	631.24
Electricity From Biogas - Production	kWh/year	640.46
Electricity From Biogas - Avoided Electricity From Grid	kWh/year	640.46
Heat From Biogas - Production	MJ/Year	46.98
Heat From Biogas - Avoided Heat From The System	MJ/Year	46.98
Stainless Steel	kg	0.26
Copper	kg	0.01
PCV	kg	0.00
Water	kg	19637.20
Replacement – Wastewater Treatment Plant	kg	4.86
Travelling Screens - Disposal	kg/year	122.49
**END-OF-LIFE STAGE**		
Recycling-Cast Iron	kg	0.09
Recycling-Steel	kg	140.22
Recycling-Copper	kg	0.01
Recycling-Aluminium	kg	0.78
Earthworks	m^3^	2.51
Recycling-Concrete	m^3^	0.90
Asphalt - Disposal	kg	0.90
Recycling - Aggregate	kg	19.38
Disposal Of Plastic	kg	4.44

Source: Own research.

### Stage 3. – Calculating individual indices of influence for inputs and outputs

The next step of the development of the LCA model was the selection of LCIA methods for the environmental evaluation of the life cycle of the system. LCIA is the third stage of LCA after the goal and scope definition and LCI. LCIA is associated with evaluating the magnitude and significance of the potential environmental impacts. To assess the environmental potential of the system of wastewater collection, transport and treatment on the environment, the IPCC method was selected and applied in the computational LCA model.

Following the next stage of the method, it is possible to calculate individual indices of influence of the system on the environment for all the inputs and outputs, considering greenhouse gas emission indices (kg CO_2_ eq).

The calculations of individual influence indices for all the used materials, energy and substances, and greenhouse gas emission into the environment were made with the IPCC method employing SimaPro 8 software with the Ecoinvent 3 database. The calculations include individual elements of the analysed system: the septic tanks, household wastewater treatment plants, sewerage system and a central wastewater treatment plant, as well as all the stages of the life cycle. Results of the life cycle-greenhouse gas emission analyses are presented in Table 5–8. The obtained indices are necessary in order to calculate the influence of the system on the environment for any quantitative or spatial configuration and for any technical solution (e.g. number and parameters of objects, material, diameter etc.) defined during Stage 1. The last step was the development of formulas for calculating the environmental life cycle impact assessment using the selected LCIA method - IPCC. The proposed LCA model was used to assess the GHG emission of the system life cycle of a wastewater collection, transport and treatment system at a national level.

**Table 5:** Results of Greenhouse Gas Emission Indices (kg CO_2_ eq/1PE) of a septic tank’s life cycle.

Carbon footprint of each input and output	Septic tanks
**CONSTRUCTION STAGE**	
Gravel	0.00281
Concrete	261.04788
Steel	2.04221
Cast Iron	1.87127
PCV	2.00685
Asphalt Rubber	0.42925
Polyester Resin	7.63944
Polyethylene	2.56799
Earthworks	0.53100
Water	0.00032
**USE STAGE**	
Diesel Fuel	0.51083
CO_2_ Emission	1.00000
CH_4_ Emission	25.00000
N_2_O Emission	300.42276
Electricity From Grid	1.16244
Electricity From Biogas - Production	0.00687
Electricity From Biogas - Avoided Electricity From Grid	−1.16251
**END-OF-LIFE STAGE**	
Recycling - Cast Iron	−1.55268
Earthworks	0.53047

Source: Own research.

**Table 6:** Results of Greenhouse Gas Emission Indices (kg CO_2_ eq/1PE) of a household wastewater treatment plant’s life cycle.

Carbon footprint of each input and output	Household Wastewater Treatment Plants
**CONSTRUCTION STAGE**	
Gravel	0.00281
Concrete	260.68373
Cement	0.87204
Stainless Steel	1.55497
Cast Iron	1.87183
Copper	1.97044
PCV	2.00647
Polyethylene	2.56809
Polypropylene	4.41391
Earthworks	0.53079
Geotextile PP	2.33141
Water	0.00032
Clay	0.00292
**USE STAGE**	
Diesel Fuel	0.51082
CO_2_ Emission	1.00000
CH_4_ Emission	24.96160
N_2_O Emission	300.42276
Electricity From Grid – Use Of Electricity By Wastewater Treatment Plants 2	19.91190
Electricity From Grid – Use Of Electricity For Sludge Treatment 2	20.76502
Electricity From Biogas - Production	0.00687
Electricity From Biogas - Avoided Electricity From Grid	−1.16258
Gravel	0.00281
Geotextile PP	2.34841
Stainless Steel	1.55813
Copper	1.90688
PCV	2.00644
**END-OF-LIFE STAGE**	
Recycling - Cast Iron	−1.54977
Recycling - Steel	−1.23392
Recycling - Copper	−1.59549
Earthworks	0.53212

Source: Own research.

**Table 7:** Results of Greenhouse Gas Emission Indices (kg CO_2_ eq/1PE) of a sewerage system’s life cycle.

Carbon footprint of each input and output	Sewerage system
**CONSTRUCTION STAGE**	
Gravel	0.00281
Concrete	261.16464
Polymer Concrete	1.53069
Stone	0.17449
Stoneware	0.14616
Steel	2.04203
Stainless Steel	2.52425
Galvanised Steel	2.70744
Cast Iron	1.87152
Copper	1.95445
PCV	2.00633
Synthetic Rubber	2.64553
Asphalt	0.42950
Polyethylene	2.56839
Earthworks	0.53100
Water	0.00032
**USE STAGE**	
Diesel Fuel	0.51083
Petrol	0.72947
Electricity From Grid	1.16246
Replacement - Sewerage System	0.08633
**END-OF-LIFE STAGE**	
Recycling - Cast Iron	−1.55289
Recycling - Steel	−2.22050
Recycling - Copper	−1.63529

Source: Own research.

**Table 8:** Results of Greenhouse Gas Emission Indices (kg CO_2_ eq/1PE) of a central wastewater treatment plant’s life cycle.

Carbon footprint of each input and output	Central wastewater treatment plant
**CONSTRUCTION STAGE**	
Concrete	261.34861
Aggregate	0.00213
Reinforcing Steel	1.44636
Stainless Steel	1.55594
Cast Iron	1.90647
Aluminium	8.52985
Copper	1.94843
Mineral Wool	1.12869
Glass fibre	2.63347
Synthetic Rubber	2.64195
Asphalt	0.42803
Polyethylene	1.92819
Polyethylene LDPE	2.16542
Plastic Foil	0.52369
Earthworks	0.53099
Water	0.00032
Organic Chemicals	1.89488
Inorganic Chemicals	1.86556
Electricity From Grid	1.18773
**USE STAGE**	
Diesel Fuel	0.51064
Petrol	0.72805
Consumables – Wastewater Treatment Plant	0.94794
CO_2_ Emission	1.00000
CH_4_ Emission	25.00042
N_2_O Emission	298.00000
Electricity From Grid	1.16240
Electricity From Biogas - Production	0.00687
Electricity From Biogas - Avoided Electricity From Grid	−1.16253
Heat From Biogas - Production	0.00068
Heat From Biogas - Avoided Heat From System	−0.14862
Water	0.00032
Replacement - Wastewater Treatment Plant	2.90813
Travelling Screens - Disposal	0.50917
**END-OF-LIFE STAGE**	
Recycling - Cast Iron	−1.54674
Recycling - Steel	−1.12791
Recycling - Copper	−1.57066
Recycling - Aluminium	−8.23304
Earthworks	0.53099
Recycling - Concrete	−261.02981
Asphalt - Disposal	2.34110
Recycling - Aggregate	−0.00213
Disposal Of Plastic	0.08968

The environmental indices for given inputs and outputs obtained during Stage 3 are used to conduct a Life Cycle Assessment of the whole system of wastewater collection, transport and treatment, following Eq. (1).

1 $${P}_{i,j,k}={M}_{i,j,k}\cdot {W}_{i,j,k,}$$

where:

P_i,j,k_ – the influence on the environment generated by used material, energy, substance or pollution emissions into the environment, for j-th element of the system at k-th life cycle stage per 1 PE,

M_i,j,k_ – the amount of used material, energy, substances and pollution emissions into the environment (for j-th element of the system and k-th life cycle stage) per 1 PE,

W_i,j,k,_ – the environmental influence index (greenhouse gas emission) for i-th type of material, substance or pollution emission into the environment (for j-th element of the system and k-th life cycle stage),

i – the type of material, substance or emission into the environment (e.g. sand, steel, PCV, electricity, pollution emission and/or gases, such as phosphorus, carbon dioxide, etc.),

j – an element of the system (septic tanks, household wastewater treatment plants, sewerage system and the central wastewater treatment plant),

k – the life cycle stage (construction, use, end-of-life).

The environmental assessment per functional unit (1 PE) was determined based on the product of individual greenhouse gas emission index and the amount of materials and substances used or emissions released into the environment during the construction, use and end-of-life stages of a given element of the system.

### Stage 4. – Calculating the influence of the system on the environment per 1 PE

The equation presented in Stage 3 (1) determines the influence on the environment attributed to one type of material, energy, substance or pollution emission into the environment.

To calculate the influence of a given element of the system on the environment per 1 PE at a given life cycle stage, considering all types of materials, energy, substances and pollution emission, the following formula has to be followed (2):

2 $$\sum _{i}{P}_{i,j,k}={P{\prime} }_{j,k}$$

where:

P_i,j,k_ – the influence on the environment generated by i-th type of material (for j-th element of the system at k-th life cycle stage) per 1 PE,

P′_j,k,_ – the influence on the environment (for j-th element of the system and at k-th life cycle stage) per 1 PE,

i – the type of material, substance or emission into the environment (e.g. sand, steel, PCV, electricity, pollution emission and/or gases, such as phosphorus, carbon dioxide, etc.),

j – an element of the system (septic tanks, household wastewater treatment plants, sewerage system and the central wastewater treatment plant),

k – the life cycle stage (construction, use, end-of-life).

The presented Eq. (2) enables the calculation of the influence of given elements of the system on a given life cycle stage. To calculate the total influence of given elements of the system on the environment throughout the life cycle (considering construction, use and end-of-life stages), the following equation must be applied (3)

3 $$\sum _{k}{P{\prime} }_{j,k}={P{\prime\prime} }_{j}$$

where:

P′_j,k,_ – the influence on the environment (for j-th element of the system and at k-th life cycle stage) per 1 PE,

P″_j,_ – the influence on the environment generated by j-th element of the system per 1 PE,

j – an element of the system (septic tanks, household wastewater treatment plants, sewerage system and the central wastewater treatment plant),

k – the life cycle stage (construction, use, end-of-life).

The equations presented in Stage 4 enable the assessment of the greenhouse gas emission for given elements of the system throughout the life cycle with reference to 1 PE.

### Stage 5. – Calculating the influence of the system of wastewater collection, transport and treatment on the environment

The next stage, following the method, concerns calculating the total influence of the system on the environment with reference to all its users (total PE). To make the calculations it is necessary to consider the total number of PE for each of the elements of the system. Equation (4) enables the calculation of the total influence, which results from using a given type of material or substance and its emissions into the environment at a given life cycle stage, on the environment:

4 $${P}_{i,j,k}\cdot {W{\prime} }_{j}={R}_{i,j,k}$$

where:

R_i,j,k_ – the total influence on the environment generated by i-th type of material (for j-th element of the system and at k-th life cycle stage),

P_i,j,k_ – the influence on the environment generated by i-th type of material (for j-th element of the system at k-th life cycle stage) per 1 PE,

W′_j_ – the total PE estimated for j-th element of the system,

i – the type of material, substance or emission into the environment (e.g. sand, steel, PCV, electricity, pollution emission and/or gases, such as phosphorus, carbon dioxide, etc.),

j – an element of the system (septic tanks, household wastewater treatment plants, sewerage system and the central wastewater treatment plant),

k – the life cycle stage (construction, use, end-of-life).

To assess the greenhouse gas emission for the life cycle of a given element of the system and a given life cycle stage, it is necessary to make calculations following this Eq. (5):

5 $$\sum _{i}{R}_{i,j,k}={R{\prime} }_{j,k}$$

where:

R_i,j,k_ – the total influence on the environment generated by i-th type of material (for j-th element of the system and at k-th life cycle stage),

R′_j,k_ – the total influence on the environment (for j-th element of the system and at k-th life cycle stage),

i – the type of material, substance or emission into the environment (e.g. sand, steel, PCV, electricity, pollution emission and/or gases, such as phosphorus, carbon dioxide, etc.),

j – an element of the system (septic tanks, household wastewater treatment plants, sewerage system and the central wastewater treatment plant),

k – the life cycle stage (construction, use, end-of-life).

To calculate the total influence on the environment generated by a given element of the system throughout the life cycle, it is necessary to follow this Eq. (6):

6 $$\sum _{k}{R{\prime} }_{j,k}={R{\prime\prime} }_{j}$$

where:

R′_j,k_ – the total influence on the environment (for j-th element of the system and at k-th life cycle stage),

R″_j_ – the total influence on the environment generated by j-th element of the system,

j – an element of the system (septic tanks, household wastewater treatment plants, sewerage system and the central wastewater treatment plant),

k – the life cycle stage (construction, use, end-of-life).

The total influence on the environment of the system of wastewater collection, transport and treatment ($R\text{'}\text{'}\text{'})\,$can be calculated as:

7 $$\sum _{j}{R{\prime\prime} }_{j}=R\prime\prime\prime $$

R″_j_ – the total influence on the environment generated by j-th element of the system,

R′″ – the total influence on the environment generated by the system,

j – an element of the system (septic tanks, household wastewater treatment plants, sewerage system and the central wastewater treatment plant).

## Results and discussion

The model enables the assessment of individual influence indices for all the materials, energy, substances and pollution emission into the environment for each life cycle stage of each element of the system and for the system itself as a whole. It is also possible to determine the aggregated index of the environmental assessment for each element of the system - septic tanks, household wastewater treatment plants, sewerage system and central wastewater treatment plant and for the system itself as a whole. Such a model enables the comparison of environmental effectiveness of the variants of the system of wastewater collection, transport and treatment, where given elements can have a different share of the whole. It enables simulations which show how modifying a given system influences the volume of the greenhouse gas emission. The model enables estimations of changes to the total influence of the system on the environment which result from changes to the share of given elements in the whole system of wastewater collection, transport and treatment.

### Carbon footprint assessment of the system of wastewater collection, transport and treatment in a city with over 50 000 inhabitants – base variant

#### Description of the system elements

There are 793 operational *septic tanks* in the analysed area of which: 567 are concrete, 155 are HDPE and 71 are GRP. The average capacity of a tank is 10 m^3^ which enables the collection of the wastewater produced by a typical household over one month. Septic tanks in the analysed city serve 2 379 PE. They are mainly located in the suburbs, in housing areas without a sewerage system. Wastewater from the tanks is transported using vacuum tankers to the wastewater treatment plant. The average distance between a tank and the wastewater treatment plant is approximately 8 kilometres.

In the city analysed there are 304 *household wastewater treatment plants*. There are 290 with septic tanks: 203 of which have a drainage field for the treated wastewater and 87 have a soakaway which fulfils the same purpose. Additionally there are 14 household wastewater treatment plants with active sludge technology: 10 with a bioreactor and a drainage field for treated wastewater, and 4 with a soakaway. Household wastewater treatment plants serve 912 PE and, like *septic tanks*, are located mainly in the suburbs, in detached housing areas without a sewerage system. Wastewater from wastewater treatment plants is discharged into the ground, and the sludge from septic tanks is transported using vacuum tankers to be treated in the central wastewater treatment plant. The average distance between a household wastewater treatment plant and the central wastewater treatment plant is approximately 8 km.

The analysed *sewerage system*, built as a blend of a gravity system and pressure system, consists of gravity sewers connected with wells where wastewater is transported with gravity flow under atmospheric pressure, as well as using wastewater pumping stations and sewer force mains which lift the level of the wastewater to the gravity sewer. After lifting, the wastewater flows due to gravity alone. The total length of the sewerage system is 237.9 km and it is built of different materials and is of different diameters. The material structure of the sewerage system network is typical for systems operating in cities of over 15 000 PE and built within the last 20 years. Modern PCV and stoneware gravity sewers are dominant (representing over 50%). The pressure sewers are made entirely of HDPE. The sewerage system is mainly located along public roads (local roads, regional roads and national roads) and seldomly on private land. Building sewerage system networks along roads means disturbing rigid pavements, e.g. asphalt. The extensive sewerage system with one wastewater treatment plant, due to the terrain conditions, requires cooperation with 49 wastewater pumping stations. The sewerage system serves 6 685 inhabitants. It enables the collection of wastewater expressed as 57 000 PE. The network transports domestic wastewater and industrial wastewater to the central wastewater treatment plant.

**Figure 4:**
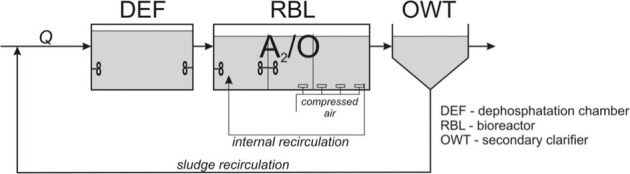
Three-stage system type A_2_/O to remove carbon, nitrogen and phosphorus Source: Own research basing on Heidrich and Witkowski (2005), Kusy and Tol (2013).

*The central wastewater treatment plant* is a facility which employs mechanical, biological and chemical processes, together with anaerobic sludge treatment. The plant is technologically divided into two parts dealing with wastewater and sludge. The wastewater part applies the classic three-stage system BARDENPHO® (Barnard Denitrification Phosphorus Removal) called A2/O (Anaerobic/Anoxic/Oxic) for the integrated removal of carbon, nitrogen and phosphorus compounds through processing active sludge in bioreactors (RBL) with plug flow (Kusy and Tol 2013). In integrated systems for dephosphatation, nitrification and denitrification, the technological process of the wastewater treatment plant includes an anoxic chamber. Figure 4 presents a general outline of the wastewater treatment process applied in the analysed wastewater treatment plant.

The analysed wastewater treatment plant is subjected to a load of contaminants expressed as 70 000 PE. The designed amount of treated wastewater discharged into a receiver, a river, are as follows: Q_maxh_ = 969 m^3^/h, Q_śrd_ = 11 622 m^3^/d, Q_maxr_ = 4 242 030 m^3^/year, maintaining acceptable physical and chemical parameters as in the regulation (ISAP 2014a).

### Application of a model to assess the carbon footprint in the life cycle of the system of wastewater collection, transport and treatment – base variant

The inputs and outputs for each element of the system, as shown in Tables 1–4, were based on the information obtained from the national systems of wastewater collection, transport and treatment. For all of the elements, input and output data sets were determined, which were indispensable for conducting the assessment of the carbon footprint based on LCA.

For all inputs and outputs identified for each element of the wastewater collection, transport and treatment system, environmental indicators were determined in relation to the reference unit.

Based on the model described above, and the data from Tables 1–4, the following indices of the carbon footprint per functional unit, i.e. 1 PE, were obtained (Tables 5–9). The results of greenhouse gas emission indices were obtained using the SimaPro software 8 and Ecoinvent 3 database with IPCC method. The results presented are split into elements of the system, life cycle stages and in total for the whole life cycle.

**Table 9:** Results of comparative analysis LCA of the system of wastewater collection, transport and treatment.

Element of system	Unit	Construction stage	Use stage	End-of-life stage	All stages, Total
Septic tanks	kg CO_2_ eq/FU	440.97	156.32	−21.68	575.61
Household wastewater treatment plants	kg CO_2_ eq/FU	292.77	251.05	−3.36	540.46
Sewerage system	kg CO_2_ eq/FU	306.91	162.64	−24.85	444.70
Central wastewater treatment plant	kg CO_2_ eq/FU	752.22	1373.61	−395.78	1 730.05
				**Total**	3 290.82

Source: Own research.

Table 9 presents the total greenhouse gas emission index of all stages of each element of the system of wastewater collection, transport and treatment: septic tank, household wastewater treatment plant, household wastewater treatment plant and central wastewater treatment plant. The greenhouse gas emission index for the analysed system of wastewater collection, transport and treatment is currently 3 290.82 kg CO_2_eq/FU (Table 9). Considering the current number of PE served in the city analysed, the total greenhouse gas emission index throughout the life cycle can be calculated. The length of a life cycle is determined by the construction stage, 30-year-long use stage, and the end-of-life stage. Table 10 presents the number of PE served.

**Table 10:** Number of PE served, current situation.

Element of system	Number of served PE
Septic tanks	2 379 PE
Household wastewater treatment plants	912 PE
Sewerage system	57 000 PE
Central wastewater treatment plant	70 000 PE

Source: Own research.

The greenhouse gas emission throughout the life cycle of the system of wastewater collection, transport and treatment in the analysed city is 148 388 532.65 kg CO_2_ eq. (Table 11). As the values of the indices of the LCA method are relative, the number itself does not provide too much information. Thus, it is necessary to conduct an analysis of the variants of the technical and functional solutions for the system to determine which changes result in a positive influence on the environment in the form of lowering the greenhouse gas emission index.

**Table 11:** Greenhouse gas emission in the life cycle of the analysed system of wastewater collection, transport and treatment – base variant.

	Unit	Septic tanks	Household wastewater treatment plants	Sewerage system	Wastewater treatment plant	Whole system
GHG emission in life cycle	kg CO_2_ eq	1 368 730	491 771.6	25 332 138	121 000 000	148 388 532.65

Source: Own research.

### Carbon footprint assessment of the system of wastewater collection, transport and treatment in a city of over 50 000 inhabitants – variant analysis

Three feasible variants of system development were analysed from a functional, technical, organizational and financial point of view. They formed the basis for the comparative analysis with the existing solution (base variant):

In *Variant 1* the existing system configuration remains unchanged, but its effectiveness changes i.e.:

- septic tanks are sealed and uncontrolled discharge of untreated wastewater from septic tanks is prevented,
- all the wastewater which requires treatment is taken to the central wastewater treatment plant, which means an increase in the number of transports with vacuum tankers,
- an increase in the use of biogas by 10%, resulting from intensifying wastewater sludge treatment processes (5%), eliminating burning biogas in a torch (also 5%) and eliminating sales of energy surplus produced from biogas to the grid (approximately 2 000 kWh).

In *Variant 2*, septic tanks are replaced with ultra-efficient household wastewater treatment plants:

- 793 septic tanks are replaced with an HWTP with a bioreactor,
- existing HWTPs with septic tanks and drainage/soakaways (290) are replaced with HWTPs with bioreactors,

In *Variant 3* septic tanks and household wastewater treatment plants are eliminated and the sewerage system network is built:

- 793 septic tanks and 304 HWTPs are eliminated,
- the sewerage system network is extended by 32.3 km to serve households which have been served by HWTPs and septic tanks. The new sewerage system is built as a gravity and pressure system (29.1 km of gravity sewer built of PCV ∅200, 3.2 km of pressure sewer built of PE ∅90÷∅110 and five wastewater pumping stations).

The results of the life cycle assessment of the system of wastewater collection, transport and treatment for each variant were presented in Table 12.

**Table 12:** Results of the life cycle assessment of the system of wastewater collection, transport and treatment for each variant.

	Unit	Septic tanks	Household wastewater treatment plants	Sewerage system	Central wastewater treatment plant	Whole system
Base variant	kg CO_2_ eq/FU	575.61	540.46	444.70	1 730.05	3 290.82
Variant 1	kg CO_2_ eq/FU	1 062.69	540.46	444.70	1 629.55	3 677.40
Variant 2	kg CO_2_ eq/FU	—	4 443.85	444.70	1 715.91	6 604.46
Variant 3	kg CO_2_ eq/FU	—	—	473.7	1 622.4	2 096.1

Source: Own research with SimaPro 8 software.

In variant 1, the analysis conducted for septic tanks shows that, with reference to a functional unit, the greenhouse gas emission increases in relation to the existing solution, by approximately 85% due to increased intensity of wastewater transport with vacuum tankers. In turn, for the central wastewater treatment plant, the greenhouse gas emission decreases by 6.2% in relation to the existing solution, as 10% more biogas is used in a wastewater treatment plant. This confirms a significant positive influence of using biogas on lowering the greenhouse gas emission index, which was identified during the environmental assessment of the central wastewater treatment plant.

The analysis of variant 2 shows that, with reference to a functional unit, the influence of an HWTP on the greenhouse gas emission increases eightfold, in comparison with the existing solution, due to the fact that the wastewater from outside the wastewater system network is treated in household wastewater treatment plants. In turn, as wastewater does not have to be transported, the individual greenhouse gas emission for the central wastewater treatment plant decreases slightly.

**Figure 5:**
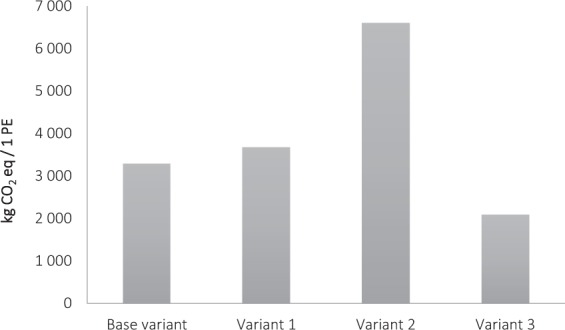
Carbon footprint comparison of variants.

For variant 3, with reference to a functional unit, due to the fact that all the wastewater is transported with the sewerage system and treated in the central wastewater treatment plant, in relation to the existing solution, the greenhouse gas emission increases by 6.5% for the sewerage system and decreases by 6.2% for the central wastewater treatment plant (Fig. 5).

To date in the literature, there has not been sufficient GHG emission analysis of the life cycle of a system of wastewater collection, transport and treatment. It is worth emphasising that this paper is the first to present a computational LCA model for calculating the life-cycle greenhouse-gas emission assessment for the identified input and output of each element of the system: septic tanks, household wastewater treatment plants, sewerage system and central wastewater treatment plant within the life cycle (construction, use, end-of-life stages). The paper presents a new computational LCA model that enables the assessment of the life cycle greenhouse gas emission for the life cycle of a wastewater collection, transport and treatment system. Application of the computational LCA model enables users to conduct environmental assessment for a whole system or each element of the system and support decision making in wastewater management.

## Sensitivity and uncertainty analysis

As part of the carbon footprint assessment model for the life cycle of a wastewater collection, transport and treatment system the sensitivity analysis of the impact of the calculation error (underestimation or overestimation) of the input data values for analysis was performed.

Sensitivity analysis in the modeling of technical objects or systems can be carried out simultaneously with the analysis of uncertainty and focuses on the analysis of the impact of the uncertainty of hypotheses adopted for modeling on the effects of the system operation^35^. It represents a tool to study how different uncertainties affect output information and whether the extent of uncertainty reduction resulting from epistemological sources can be determined^36^. Uncertainty may result from many sources, e.g. input data errors, model parameter evaluation errors or alternative object structure models. As a result, the uncertainty of the parameters at the output of the model subject to uncertainty analysis is obtained. However, the relative significance of individual causes of uncertainty is subject to sensitivity analysis.

The purpose of the sensitivity analysis in this paper was to examine the impact of all input factors used to model the life cycle assessment of individual elements of the wastewater collection, transport and treatment system and to indicate which factors may have a significant impact on the model results and the system’s carbon footprint and to what extent.

The sensitivity and uncertainty analysis was conducted taking into account the following assumptions and methodological steps:

1. The calculation model changed the value of each factor at each stage of the life cycle and for each of the four elements of the system within +/−30% (in total over 200 factors at 3 stages of the life cycle and 4 elements of the system.
2. The increased value in each factor change was assumed in 10% increments, i.e. −30%, −20%, −10%, +10%, +20%, +30% (6 change scenarios).
3. If the change in the result of the calculated GHG emission for a given factor at a given stage of the life cycle for any value of the factor change increase was at least 0.1%, then the impact of changing this factor on the entire life cycle of a given element was examined.
4. Over 1,200 calculations were carried out for the abovementioned assumptions.
5. The tables and charts present only those factors whose sensitivity can be defined as noticeable for the entire life cycle of a given element, i.e. a minimum of 0.1%

**Figure 6:**
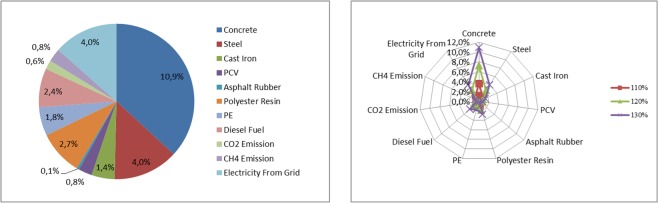
Sensitivity model of the carbon footprint assessment for factors related to septic tanks Source: Own research.

Table 13 presents the sensitivity of the amount of the greenhouse gases emitted throughout the entire life cycle for a given type of system element, i.e. septic tanks, depending on changes in the value of factors analyzed in the assessment model. Figure 6 presents a graphic illustration of the sensitivity of the model to the most important factors regarding septic tanks.

**Table 13:** Results of sensitivity analysis for septic tanks throughout the entire life cycle.

The value increase of the factor change	70%	80%	90%	100%	110%	120%	130%
**CONSTRUCTION STAGE**							
Concrete	−10,9%	−7,2%	−3,6%	0,0%	3,6%	7,2%	10,9%
Steel	−4,0%	−2,7%	−1,3%	0,0%	1,3%	2,7%	4,0%
Cast Iron	−1,4%	−0,9%	−0,5%	0,0%	0,5%	0,9%	1,4%
PCV	−0,8%	−0,6%	−0,3%	0,0%	0,3%	0,6%	0,8%
Asphalt Rubber	−0,1%	−0,1%	0,0%	0,0%	0,0%	0,1%	0,1%
Polyester Resin	−2,7%	−1,8%	−0,9%	0,0%	0,9%	1,8%	2,7%
PE	−1,8%	−1,2%	−0,6%	0,0%	0,6%	1,2%	1,8%
**USE STAGE**							
Diesel Fuel	−2,4%	−1,6%	−0,8%	0,0%	0,8%	1,6%	2,4%
CO2 Emission	−0,6%	−0,4%	−0,2%	0,0%	0,2%	0,4%	0,6%
CH4 Emission	−0,8%	−0,5%	−0,3%	0,0%	0,3%	0,5%	0,8%
Electricity From Grid	−4,0%	−2,6%	−1,3%	0,0%	1,3%	2,6%	4,0%

Source: Own research.

The change in the use of concrete has the greatest impact on greenhouse gas emissions at the construction stage of septic tanks. Underestimation or overestimation of the amount of concrete used for tank construction by +/− 30% results in a change in the amount of greenhouse gases by +/− 10.9%. In addition, the use of steel and polyester resins show significant sensitivity to GHG emissions. However, at the stage of operation of septic tanks, the change in energy consumption from the network to treat transported sewage and diesel oil consumption during sewage disposal has the largest impact on greenhouse gas emissions. A +/− 30% change in energy consumption causes a sensitivity of +/− 4.0% for greenhouse gas emissions, while a change in diesel fuel consumption to the same extent affects a +/− 2.4% change in greenhouse gas emissions.

**Figure 7:**
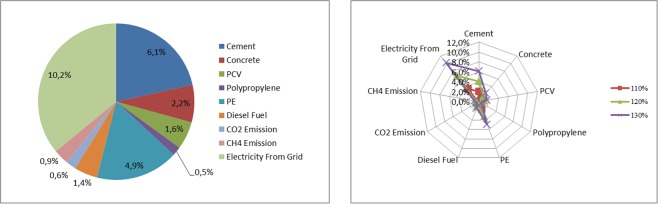
Sensitivity model of carbon footprint assessment for factors regarding household wastewater treatment plants. Source: Own research.

Table 14 presents the sensitivity of the amount of emitted greenhouse gases throughout the life cycle for a given type of system element, i.e. household wastewater treatment plants, depending on changes in the value of factors analyzed in the assessment model. Figure 7 presents a graphic illustration of the sensitivity of the model to the most important factors regarding household wastewater treatment plants.

**Table 14:** Results of sensitivity analysis for individual wastewater treatment plants throughout the entire life cycle.

The value increase of the factor change	70%	80%	90%	100%	110%	120%	130%
**CONSTRUCTION STAGE**							
Cement	−6,1%	−4,1%	−2,0%	0,0%	2,0%	4,1%	6,1%
Concrete	−2,2%	−1,5%	−0,7%	0,0%	0,7%	1,5%	2,2%
PCV	−1,6%	−1,1%	−0,5%	0,0%	0,5%	1,1%	1,6%
Polypropylene	−0,5%	−0,3%	−0,2%	0,0%	0,2%	0,3%	0,5%
PE	−4,9%	−3,2%	−1,6%	0,0%	1,6%	3,2%	4,9%
**USE STAGE**							
Diesel Fuel	−1,4%	−0,9%	−0,5%	0,0%	0,5%	0,9%	1,4%
CO2 Emission	−0,6%	−0,4%	−0,2%	0,0%	0,2%	0,4%	0,6%
CH4 Emission	−0,9%	−0,6%	−0,3%	0,0%	0,3%	0,6%	0,9%
Electricity From Grid	−10,2%	−6,8%	−3,4%	0,0%	3,4%	6,8%	10,2%

Source: Own research.

The exploitation stage of household wastewater treatment plants is when greenhouse gas emission levels are most sensitive. The change in energy consumption from the network has the greatest impact on the treatment of sludge taken from household wastewater treatment plants to a central wastewater treatment plant. A +/− 30% change in energy consumption results in a +/− 10.2% change in greenhouse gas emissions. The construction stage of household wastewater treatment plants, in terms of cement and PE use, shows significant sensitivity to greenhouse gas emissions. Underestimation or overestimation of the amount of cement for the construction of household wastewater treatment plant by +/− 30% causes a change in the amount of greenhouse gases by +/− 6.1%, while in the case of PE this change is +/− 4.9%. In addition, the use of concrete (+/−2.2%) has a large impact on GHG emissions.

**Figure 8:**
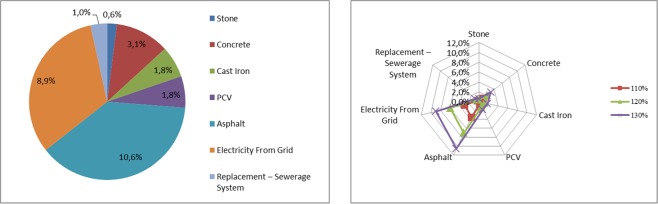
Sensitivity model of carbon footprint assessment for factors regarding a sewerage system. Source: Own research.

Table 15 presents sensitivity to the amount of emitted greenhouse gases throughout the entire life cycle for a sewerage system, depending on the changes in the value of factors analyzed in the assessment model for this element of the system. Figure 8 presents a graphic illustration of the sensitivity of the model to the most important factors related to the life cycle of the sewerage system.

**Table 15:** Results of sensitivity analysis for a sewerage system throughout its life cycle.

The value increase of the factor change	70%	80%	90%	100%	110%	120%	130%
**CONSTRUCTION STAGE**							
Stone	−0,6%	−0,4%	−0,2%	0,0%	0,2%	0,4%	0,6%
Concrete	−3,1%	−2,1%	−1,0%	0,0%	1,0%	2,1%	3,1%
Cast Iron	−1,8%	−1,2%	−0,6%	0,0%	0,6%	1,2%	1,8%
PCV	−1,8%	−1,2%	−0,6%	0,0%	0,6%	1,2%	1,8%
Asphalt	−10,6%	−7,1%	−3,5%	0,0%	3,5%	7,1%	10,6%
**USE STAGE**							
Electricity From Grid	−8,9%	−6,0%	−3,0%	0,0%	3,0%	6,0%	8,9%
Replacement – Sewerage System	−1,0%	−0,6%	−0,3%	0,0%	0,3%	0,6%	1,0%

Source: Own research.

Greenhouse gas emissions show the greatest sensitivity during the construction stage of a sewerage system in the scope of bituminous mass use (asphalt) during reconstruction of road surfaces and production of concrete. Underestimation or overestimation of the amount of bituminous mass by +/− 30% leads to a change in the amount of greenhouse gases by +/− 10.6%, while in the case of concrete production this change is +/− 3.1%. Other parameters no longer have such a great impact. However, at the stage of the operation of the sewerage system, the change in energy consumption at sewage pumping stations is most likely to lead to changes in greenhouse has emissions. A +/− 30% change in energy consumption shows a sensitivity of greenhouse gas emissions of +/− 8.9%.

**Figure 9:**
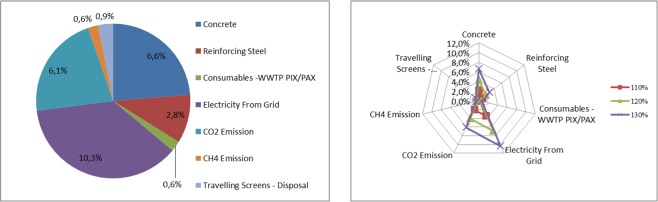
Sensitivity model of carbon footprint assessment for factors related to central wastewater treatment plant Source: Own research.

Table 16 presents sensitivity of the amount of emitted greenhouse gases throughout the life cycle for a central wastewater treatment plant, depending on changes in the value of factors analyzed in the assessment model for this element of the system. Figure 9 presents a graphic illustration of the sensitivity of the model to the most important factors related to the life cycle of the central wastewater treatment plant.

**Table 16:** Results of the sensitivity analysis for a central wastewater treatment plant throughout its whole life cycle.

The value increase of the factor change	70%	80%	90%	100%	110%	120%	130%
**CONSTRUCTION STAGE**							
Concrete	−6,6%	−4,4%	−2,2%	0,0%	2,2%	4,4%	6,6%
Reinforcing Steel	−2,8%	−1,9%	−0,9%	0,0%	0,9%	1,9%	2,8%
**USE STAGE**							
Consumables -WWTP PIX/PAX	−0,6%	−0,4%	−0,2%	0,0%	0,2%	0,4%	0,6%
Electricity From Grid	−10,3%	−6,9%	−3,4%	0,0%	3,4%	6,9%	10,3%
CO2 Emission	−6,1%	−4,1%	−2,0%	0,0%	2,0%	4,1%	6,1%
CH4 Emission	−0,6%	−0,4%	−0,2%	0,0%	0,2%	0,4%	0,6%
Travelling Screens - Disposal	−0,9%	−0,6%	−0,3%	0,0%	0,3%	0,6%	0,9%

Source: Own research.

Greenhouse gas emissions show greatest sensitivity during the exploitation stage, which is associated with a central wastewater treatment plant, in particular in terms of energy consumption from the network and direct CO2 emissions from the central wastewater treatment plant facility to the atmosphere. A +/− 30% change in energy consumption causes a sensitivity of +/− 10.3% in greenhouse gas emissions. In the case of direct CO2 emissions into the atmosphere from the central wastewater treatment plant, a change of +/− 30% on the life cycle scale of the treatment plant causes a change in overall emissions of +/− 6.1%.

Significant sensitivity connected to this element of the system is also associated with the stage of construction of the central wastewater treatment plant. Underestimating or overestimating the amount of concrete by +/− 30% results in a change in the amount of greenhouse gases by +/− 6.6%, while in the case of reinforcing steel production this change is +/− 2.8%.

Other parameters do not have such a significant impact on the final balance of greenhouse gas emissions from the central wastewater treatment plant.

The conducted sensitivity analysis showed that it is possible to indicate factors, whose values are adopted in the model, which are significant for the simulation results of the model. Each of the system elements is sensitive to several important input factors related to the use of raw materials in the life cycle, at a level of up to approximately 10%. It is important to indicate the stages of construction of all the system components where factors such as the use of concrete, steel, cement, polyethylene and asphalt determine the highest greenhouse gas emissions and a change in their value of up to 30% may increase or decrease GHG emissions throughout the entire life cycle from 4.0% to up to approximately 10%.

GHG emission sensitivity at the system exploitation stage is mainly related to energy consumption and in this respect the model shows a significant relationship where energy consumption for household wastewater treatment plants, sewerage system and central wastewater treatment plant changes up to 30%, GHG emissions change by 9%–11%. It should be highlighted that the vast majority of input parameters in the model, in the form of factors which show the highest sensitivity, can be accurately measured at the stage of designing work and operational measurements, therefore, it can be concluded that uncertainty in this respect does not significantly distort the results of the assessment.

The sensitivity of changes in GHG emissions to input/output factors at the decommissioning stage of each of the system components is negligible.

## Conclusions

The developed method enables the assessment of the influence of each element of wastewater collection, transport and treatment system throughout its life cycle or at any of its stages. The assessment can be conducted for the current state of a system, for a newly-planned system or the expansion of a system. It enables the comparative analyses of different system configurations. The assessment is conducted with reference to a functional unit expressed as 1 PE and for the whole system, considering the influence of all the system elements on greenhouse gas emission.

The application of a functional unit, against which the environmental assessment is conducted for the whole system, i.e. 1 PE, is a novel approach which enables a universal reference level to be obtained whilst comparing elements of the system and other systems. It was observed that the central wastewater treatment plant has the highest greenhouse gas emission, while the sewerage system has the lowest. During the construction stage, the highest greenhouse gas emission was observed for three elements of the system, but not the central wastewater treatment plant, where the greenhouse gas emission was the highest at the use stage. The analyses showed that using biogas to produce electricity instead of using electricity from the grid has a lot of benefits for the environment. Every element of the system where biogas was applied showed a decrease in greenhouse gas emission. Analyses show that that recycling at the end-of-life stage is an environmental benefit for greenhouse gas emission. Comparative analyses conducted for different variants of technical solutions showed that the use of energy at the use stage of a wastewater treatment plant determines the influence of the whole system on greenhouse gas emission, and the most effective decrease in greenhouse gas emission in the whole system is obtained by increasing the production of electricity from biogas. An increase in the use of biogas to produce electricity by 10% in the wastewater treatment plant enables the lowering of emissions in the whole system by 3.65%. One important aspect is the comparison of the variants of operating septic tanks and HWTPs (scattered system) and the sewerage system network with the central wastewater treatment plant (centralised system), which shows that, with reference to environmental benefits associated with lowering greenhouse gas emission, the sewerage system network is a better solution than septic tanks and HWTPs. The conducted sensitivity analysis showed that it is possible to indicate the factors, whose values are adopted in the model, which are significant for the results of the assessment, which can be the basis for improving the design and operational aspects of the system to reduce greenhouse gas emissions. It is an important conclusion in the search for solutions to lower carbon footprint in wastewater management, especially in those areas where there are dilemmas concerning which solution is better from an economic and an environmental point of view, e.g. in suburbs or housing areas.

The research problem addressed is significant with regards to the European Commission’s guidelines and the new challenges of the circular economy. Therefore, the results obtained and the assessment method may be a practical tool for the assessment of whether the aforementioned guidelines are met in wastewater management. Both the model itself and the detailed data collected for the city system of differentiated types of housing and over 50 000 PE will undoubtedly be useful in further research.
